# Supplementary material for: Modeling glioblastoma heterogeneity as a dynamic network of cell states
Source: Mol Syst Biol. 2021 Sep 16;17(9):e10105. doi: 10.15252/msb.202010105 (PMC8444284; doi:10.15252/msb.202010105)
Supplement: Supplementary file 6 — Source Data for Figure 5 [file MSB-17-e10105-s004.zip › Figure5A_sourcedata/GSEA_3071/hallmarks_stateVI.GseaPreranked.1623742557031/HALLMARK_IL6_JAK_STAT3_SIGNALING.html]

Details for gene set HALLMARK\_IL6\_JAK\_STAT3\_SIGNALING[GSEA]

|  || Dataset | state63071 |
| Phenotype | NoPhenotypeAvailable |
| Upregulated in class | na\_neg |
| GeneSet | HALLMARK\_IL6\_JAK\_STAT3\_SIGNALING |
| Enrichment Score (ES) | -0.24749346 |
| Normalized Enrichment Score (NES) | -0.84070706 |
| Nominal p-value | 0.63674617 |
| FDR q-value | 0.7676378 |
| FWER p-Value | 1.0 |
Table: GSEA Results Summary

  

Fig 1: Enrichment plot: HALLMARK\_IL6\_JAK\_STAT3\_SIGNALING      
 Profile of the Running ES Score & Positions of GeneSet Members on the Rank Ordered List

  

| PROBE | GENE SYMBOL | GENE\_TITLE | RANK IN GENE LIST | RANK METRIC SCORE | RUNNING ES | CORE ENRICHMENT || 1 | JUN |  |  | 321 | 0.527 | -0.0272 | No |
| 2 | CXCL3 |  |  | 359 | 0.508 | 0.0219 | No |
| 3 | SOCS1 |  |  | 489 | 0.458 | 0.0398 | No |
| 4 | PIM1 |  |  | 582 | 0.430 | 0.0646 | No |
| 5 | IRF1 |  |  | 771 | 0.382 | 0.0572 | No |
| 6 | SOCS3 |  |  | 775 | 0.381 | 0.1008 | No |
| 7 | PTPN2 |  |  | 1044 | 0.338 | 0.0663 | No |
| 8 | BAK1 |  |  | 1249 | 0.310 | 0.0461 | No |
| 9 | HAX1 |  |  | 1423 | 0.289 | 0.0321 | No |
| 10 | STAT3 |  |  | 1861 | -0.259 | -0.0584 | No |
| 11 | IFNAR1 |  |  | 1897 | -0.263 | -0.0373 | No |
| 12 | STAT1 |  |  | 2199 | -0.314 | -0.0839 | No |
| 13 | STAT2 |  |  | 2345 | -0.342 | -0.0840 | No |
| 14 | CNTFR |  |  | 2748 | -0.440 | -0.1437 | No |
| 15 | TNFRSF21 |  |  | 3125 | -0.600 | -0.1774 | Yes |
| 16 | ITGA4 |  |  | 3132 | -0.610 | -0.1079 | Yes |
| 17 | CD9 |  |  | 3445 | -0.995 | -0.0779 | Yes |
| 18 | A2M |  |  | 3503 | -1.122 | 0.0373 | Yes |
Table: GSEA details [plain text format]

  

Fig 2: HALLMARK\_IL6\_JAK\_STAT3\_SIGNALING: Random ES distribution      
 Gene set null distribution of ES for **HALLMARK\_IL6\_JAK\_STAT3\_SIGNALING**
